# New cryptic species of the ‘*revolutum*’ group of *Echinostoma* (Digenea: Echinostomatidae) revealed by molecular and morphological data

**DOI:** 10.1186/1756-3305-6-64

**Published:** 2013-03-13

**Authors:** Simona Georgieva, Christian Selbach, Anna Faltýnková, Miroslava Soldánová, Bernd Sures, Karl Skírnisson, Aneta Kostadinova

**Affiliations:** 1 Institute of Parasitology, Biology Centre of the Academy of Sciences of the Czech Republic, Branišovská 31, České Budějovice, 370 05, Czech Republic; 2 Department of Aquatic Ecology and Centre for Water and Environmental Research (ZWU), University of Duisburg-Essen, Universitätsstraße 5, Essen D-45141, Germany; 3 Laboratory of Parasitology, Institute for Experimental Pathology, University of Iceland, Reykjavik, Keldur, 112, Iceland; 4 Institute of Biodiversity and Ecosystem Research, Bulgarian Academy of Sciences, 2 Gagarin Street, Sofia, 1113, Bulgaria

**Keywords:** *Radix auricularia*, *Radix peregra*, *Stagnicola palustris*, *Echinostoma*, Cryptic species, Europe

## Abstract

**Background:**

The digenean species of *Echinostoma* (Echinostomatidae) with 37 collar spines that comprise the so-called ‘*revolutum*’ species complex, qualify as cryptic due to the interspecific homogeneity of characters used to differentiate species. Only five species were considered valid in the most recent revision of the group but recent molecular studies have demonstrated a higher diversity within the group. In a study of the digeneans parasitising molluscs in central and northern Europe we found that *Radix auricularia*, *R. peregra* and *Stagnicola palustris* were infected with larval stages of two cryptic species of the ‘*revolutum*’ complex, one resembling *E. revolutum* and one undescribed species, *Echinostoma* sp. IG. This paper provides morphological and molecular evidence for their delimitation.

**Methods:**

Totals of 2,030 *R. auricularia,* 357 *R. peregra* and 577 *S. palustris* were collected in seven reservoirs of the River Ruhr catchment area in Germany and a total of 573 *R. peregra* was collected in five lakes in Iceland. Cercariae were examined and identified live and fixed in molecular grade ethanol for DNA isolation and in hot/cold 4% formaldehyde solution for obtaining measurements from fixed materials. Partial fragments of the mitochondrial gene nicotinamide adenine dinucleotide dehydrogenase subunit 1 (*nad*1) were amplified for 14 isolates.

**Results:**

Detailed examination of cercarial morphology allowed us to differentiate the cercariae of the two *Echinostoma* spp. of the ‘*revolutum*’ species complex. A total of 14 partial *nad*1 sequences was generated and aligned with selected published sequences for eight species of the ‘*revolutum*’ species complex. Both NJ and BI analyses resulted in consensus trees with similar topologies in which the isolates from Europe formed strongly supported reciprocally monophyletic lineages. The analyses also provided evidence that North American isolates identified as *E. revolutum* represent another cryptic species of the ‘*revolutum*’ species complex.

**Conclusion:**

Our findings highlight the need for further analyses of patterns of interspecific variation based on molecular and morphological evidence to enhance the re-evaluation of the species and advance our understanding of the relationships within the ‘*revolutum*’ group of *Echinostoma*.

## Background

The digenean species of *Echinostoma* Rudolphi, 1809 (Echinostomatidae) with 37 collar spines that comprise the so-called *Echinostoma* ‘*revolutum*’ complex, qualify as cryptic (*sensu* Bickford *et al.*[1]; see also Pérez-Ponce de León and Nadler [2] for a recent review) due to the interspecific homogeneity of characters used to differentiate species. Only five species, the Eurasian *Echinostoma revolutum* (Frölich, 1802), *E. echinatum* (Zeder, 1803) and *E. jurini* (Skvortsov, 1924), the North American *E. trivolvis* (Cort, 1914) and the African *E. caproni* Richard, 1964, were considered valid in the most recent revision of the group using for species delimitation a single morphological feature of the larval stages (the number of pores of the para-oesophageal gland-cells in the cercaria), the specificity towards the first intermediate host (at the familial level), the ability to infect avian or mammalian hosts (or both) and geographical range on a global scale (continents) [3-5] (but see Kostadinova and Gibson [6] for a critical review). It is worth noting that *E. echinatum* has not been formally described and justified in a taxonomic publication and is not recognised as valid [see 6 for details]. However, recent molecular studies have demonstrated a higher diversity within the ‘*revolutum*’ species complex. Thus one African species, *Echinostoma deserticum* Kechemir et al., 2002, and a yet unidentified species from New Zealand were distinguished based on molecular data [7] (see also [8]), and *E. trivolvis* was found to represent a species complex [9]. Additional data on the geographical distribution of the *Echinostoma* spp. have also been obtained. *E. revolutum* was recorded in Australia [7] and North America [10,11], *Echinostoma paraensei* Lie & Basch, 1967 in Australia and South America [7], and *E*. cf. *robustum* in North and South America [11].

The pioneer molecular studies, predominantly based on laboratory strains, have revealed that the mitochondrial *nad*1 gene provides a better resolution for investigating relationships within the problematic *Echinostoma* ‘*revolutum*’ species complex in comparison with the nuclear rRNA spacers and the mitochondrial *cox*1 gene [12,13]. The subsequent DNA-based studies [7,9-11,14] have provided a framework for investigating genetic in natural *Echinostoma* spp. populations and revealed novel data on the cryptic variation, identification and geographical distribution of the species of the ‘*revolutum*’ complex.

However, in contrast with the wealth of sequences gathered recently from North America, which have revealed high diversity (six cryptic lineages) within the ‘*revolutum*’ complex of *Echinostoma*[9,11], data from European natural populations are virtually lacking. Thus, of the eight species described and/or recorded from Europe, *i.e. E. revolutum*, *E*. *paraulum* Dietz, 1909, *E. jurini* (Skvortsov, 1924), *E. miyagawai* Ishii, 1932, *E. robustum* Yamaguti, 1935, *E*. *bolschewense* (Kotova, 1939), *E. nordiana* (Baschkirova, 1941), *E. friedi* Toledo et al., 2000 [3,5,15-22], sequence data are available only for *E. revolutum*[7,12-14] and *E. friedi* (GenBank AJ564379).

In a study of the digeneans parasitising molluscs in central and northern Europe we found that *Radix auricularia* (Linnaeus, 1758), *Radix peregra* (Müller, 1774) and *Stagnicola palustris* (Müller, 1774) were infected with larval stages of two species of the *Echinostoma* ‘*revolutum*’ complex of cryptic species, one resembling *E. revolutum* sensu stricto (s.s.) and one undescribed species (see also [23]). Here we describe the cercariae of these two species and provide morphological and molecular evidence for their delimitation. Further, we extend the approaches of Morgan and Blair [7,13], Kostadinova *et al.*[14] and Detwiler *et al.*[11] to the relationships within the ‘*revolutum*’ species complex inferred from the *nad*1 gene with the newly-generated sequence data from natural infections in snails in Europe. Phylogenetic analyses revealed the presence of additional cryptic lineages of the *Echinostoma* ‘*revolutum*’ species complex.

## Methods

### Sample collection

Totals of 2,030 *R. auricularia,* 357 *R. peregra* and 577 *S. palustris* were collected during 2009–2012 in seven reservoirs of the River Ruhr catchment area (North Rhine-Westphalia, Germany): Baldeneysee (51°24^′^20.08"N, 7°2^′^22.47"E); Harkortsee (51°23^′^40.56"N, 7°24^′^8.27"E); Hengsteysee (51°24^′^52.17"N, 7°27^′^42.55"E); Hennetalsperre (51°19^′^50.97"N, 8°15^′^46.82"E); Kemnader See (51°25^′^19.05"N, 7°15^′^43.07"E); Sorpetalperre (51°20^′^ 15.01"N, 7°56^′^46.18"E); and Versetalsperre (51°10^′^55.71"N, 7°40^′^57.12"E). Seven distinct samples of *R. peregra* (a total of 573 snails) were collected in five localities in Iceland: Lakes Family Park (64°08^′^15"N, 21°52^′^03"W) and Nordic House (64°08^′^19"N, 21°56^′^45"W) in Reykjavik; Opnur (63°58^′^43"N, 21°10^′^37"W); Raudavatn (64°05^′^35"N, 21°47^′^14"W); and Helgavogur, Lake Myvatn (65°38^′^04"N, 16°55^′^28"W) in May and August 2012. Snails were collected randomly with a strainer or picked by hand from stones and floating vegetation along the shore at several sampling sites at each reservoir. In the laboratory, snails were labelled and placed individually into beakers with a small amount of lake water, and kept under a light source for up to 5 days to stimulate emergence of cercariae. Thereafter, snails were measured, dissected and examined for prepatent infections.

### Morphological data

Cercariae were examined and identified live using the data from the keys of Faltýnková *et al.*[24,25] and other relevant primary sources [3,18-22]. Digital photographs of live cercariae (and rediae) were taken with a digital camera of an Olympus BX51 microscope. Vital stains (Neutral Red and Nile Blue sulphate) were used for visualisation of the para-oesophageal gland-cells of the cercariae. Measurements (in micrometres) were taken from the digital images with the aid of QuickPHOTO CAMERA 2.3 image analysis software or the program ImageJ [26]. Upon preliminary identification, two samples of cercariae (rediae) per isolate were fixed: (i) in molecular grade ethanol for DNA isolation and sequencing; and (ii) in hot/cold 4% formaldehyde solution for obtaining measurements from fixed materials. Snails were identified using Glöer [27]. Although *R. peregra* and *R. ovata* (Draparnaud, 1805) have recently been treated as junior synonyms of *R. balthica* (Linnaeus, 1758) we used the name *R. peregra* following the molecular studies of Bargues *et al.*[28] and Huňová *et al.*[29] which provide sequences for snails sampled in both central Europe and Iceland.

### Molecular data

Total genomic DNA was isolated from ethanol-fixed single rediae and/or 10–50 pooled cercariae obtained from a single snail individual by placing the samples in 200 μL of a 5% suspension of deionised water and Chelex® containing 0.1 mg/mL proteinase K, followed by incubation at 56°C for 3 h, boiling at 90°C for 8 min, and centrifugation at 14,000 g for 10 min. Polymerase chain reaction (PCR) amplifications of partial fragments of the mitochondrial gene nicotinamide adenine dinucleotide dehydrogenase subunit 1 (*nad*1) were performed in 25 μl reactions using Ready-To-Go-PCR Beads (GE Healthcare, UK) containing ~2.5 units of puReTaq DNA polymerase, 10 mM Tris–HCl (pH 9.0), 50 mM KCl, 1.5 mM MgCl_2_, 200 mM of each dNTP and stabilisers including BSA, 10 mM of each PCR primer, and 50 ng of template DNA. The following PCR primers were used: forward NDJ11 (equivalent to JB11 in [13]) 5^′^-AGA TTC GTA AGG GGC CTA ATA-3^′^ and reverse NDJ2a: 5^′^-CTT CAG CCT CAG CAT AAT-3^′^[14]. The thermocycling profile comprised initial denaturation at 95°C for 5 min, followed by 35 cycles with 30 s denaturation at 94°C, 20 s primer annealing at 48°C, and 45 s at 72°C for primer extension, with a final extension step of 4 min at 72°C.

PCR amplicons were purified using Qiagen QIAquick™ PCR Purification Kit (Qiagen Ltd, UK) and sequenced directly for both strands using the PCR primers. Sequencing was performed on an ABI Prism 3130xl automated sequencer using ABI Big Dye chemistry (ABI Perkin-Elmer, UK) according to the manufacturer’s protocol. Contiguous sequences were assembled and edited using MEGA v5 [30] and submitted to GenBank (accession numbers shown in Table 1).

**Table 1:** **List of species/isolates of the ‘ *revolutum *’ species complex used in this study, their hosts, localities and GenBank accession numbers**

**Species**	**Host**	**Locality**	**Accession no.**	**Reference**
***Echinostoma *sp. IG**	*Radix peregra *(isolate RPI1)	Nordic House (Iceland)	KC618448	Present study
***Echinostoma *sp. IG**	*Radix auricularia *(isolate RAG1)	Hengsteysee (Germany)	KC618449	Present study
***Echinostoma *sp. IG**	*Radix auricularia *(isolate RAG2)	Hengsteysee (Germany)	KC618450	Present study
***Echinostoma revolutum***	*Radix peregra *(isolate RPI2)	Lake Myvatn (Iceland)	KC618451	Present study
***Echinostoma revolutum***	*Radix peregra *(isolate RPI3)	Lake Myvatn (Iceland)	KC618452	Present study
***Echinostoma revolutum***	*Radix peregra *(isolate RPI4)	Lake Myvatn (Iceland)	KC618453	Present study
***Echinostoma revolutum***	*Stagnicola palustris *(isolate SPG1)	Hengsteysee (Germany)	KC618454	Present study
***Echinostoma revolutum***	*Radix auricularia *(isolate RAG3)	Hennetalsperre (Germany)	KC618455	Present study
***Echinostoma revolutum***	*Radix auricularia *(isolate RAG4)	Hennetalsperre (Germany)	KC618461	Present study
***Echinostoma revolutum***	*Radix peregra *(isolate RPG1)	Hennetalsperre (Germany)	KC618456	Present study
***Echinostoma revolutum***	*Radix peregra *(isolate RPG2)	Hennetalsperre (Germany)	KC618457	Present study
***Echinostoma revolutum***	*Radix peregra *(isolate RPG3)	Hennetalsperre (Germany)	KC618458	Present study
***Echinostoma revolutum***	*Radix peregra *(isolate RPG4)	Hennetalsperre (Germany)	KC618460	Present study
***Echinostoma revolutum***	*Radix peregra *(isolate RPG5)	Hennetalsperre (Germany)	KC618459	Present study
***Echinostoma caproni***	na	Cameroon	AF025838	Morgan & Blair [7,13]
***Echinostoma caproni***	na	Madagascar, Egypt	AF025837	Morgan & Blair [7,13]
***Echinostoma caproni***	*Rattus norvegicus*	Cairo (Egypt)	AJ564378	Marcilla et al. (unpublished)
E. deserticum^*^	na	Niger	AF025836	Morgan & Blair [7,13]
***Echinostoma *cf. *friedi***	*Planorbis* sp.	Wales (UK)	AY168937	Kostadinova *et al*. [14]
***Echinostoma friedi***	*Mesocricetus auratus *(exp.)	Pons, Valencia (Spain)	AJ564379	Marcilla *et al. *(unpublished)
***Echinostoma paraensei***	na	Brazil	AF025834	Morgan & Blair [7,13]
***Echinostoma revolutum***	*Radix peregra*/*Columba livia *(exp.)	Bulgaria	AY168933	Kostadinova *et al.*[14]
***Echinostoma revolutum***	*Lymnaea elodes*/*Gallus gallus *(exp.)	Shock Lake, Indiana (USA)	GQ463082	Detwiler *et al*. [11]
***Echinostoma revolutum***	*Lymnaea elodes*	Pond A, Indiana (USA)	GQ463088	Detwiler *et al*. [11]
***Echinostoma revolutum***	*Lymnaea elodes*	Pond A, Indiana (USA)	GQ463090	Detwiler *et al*. [11]
***Echinostoma revolutum***	*Lymnaea elodes*	Pond A, Indiana (USA)	GQ463086	Detwiler *et al*. [11]
***Echinostoma revolutum***	*Lymnaea elodes*	Shock Lake, Indiana (USA)	GQ463084	Detwiler *et al.*[11]
***Echinostoma revolutum***	*Ondatra zibethicus*	Virginia (USA)	JQ670862	Detwiler *et al*. [11]
***Echinostoma revolutum***	na	“Germany, Europe”	AF025832	Morgan & Blair [7,13]
Echinostoma robustum^**^	*Lymnaea elodes*	Minnesota (USA)	GQ463054	Detwiler *et al.*[11]
Echinostoma robustum^**^	*Biomphalaria glabrata*/*G. gallus *(exp.)	Brazil	GQ463055	Detwiler *et al*. [11]
Echinostoma robustum^**^	*Lymnaea elodes*	Pond A, Indiana (USA)	GQ463053	Detwiler *et al.*[11]
***Echinostoma trivolvis***	*Ondatra zibethicus*	Virginia (USA)	JQ670860	Detwiler *et al*. [9]
***Echinostoma trivolvis***	*Ondatra zibethicus*	Virginia (USA)	JQ670852	Detwiler *et al*. [9]
***Echinostoma trivolvis***	*Ondatra zibethicus*	Virginia (USA)	JQ670854	Detwiler *et al*. [9]
***Echinostoma trivolvis***	*Ondatra zibethicus*	Virginia (USA)	JQ670858	Detwiler *et al*. [9]
***Echinostoma trivolvis***	*Ondatra zibethicus*	Virginia (USA)	JQ670856	Detwiler *et al*. [9]
***Echinoparyphium recurvatum***	*Radix peregra*	Wales (UK)	AY168944	Kostadinova *et al.*[14]
***Echinoparyphium aconiatum***	*Lymnaea stagnalis*	Finland	AY168945	Kostadinova *et al*. [14]

^*^ Syn. *Echinostoma *sp. I Africa of Morgan and Blair [17,13]; ^**^sensu Detwiler *et al*. [11].

Newly-generated and published *nad*1 sequences for *Echinostoma* spp. (see Table 1 for details) were aligned using Clustal W implemented in MEGA v5 with reference to the amino acid translation, using the echinoderm and flatworm mitochondrial code [31]. Species boundaries were assessed via neighbour-joining (NJ) analyses of Kimura-2-parameter distances using MEGA v5 (nodal support estimated using 1,000 bootstrap resamplings) and Bayesian inference (BI) analysis using MrBayes 3.2 [32,33]. The best-fitting model of nucleotide substitution estimated prior to BI analysis using jModelTest 2.1 [34] was the Hasegawa-Kishino-Yano model including estimates of invariant sites and among-site rate heterogeneity (HKY + I + G).

BI log-likelihoods were estimated with default prior probabilities and likelihood model settings (nst = 2; rates = invgamma; ngammacat = 4) over 10^6^ generations via 4 simultaneous Markov Chain Monte Carlo chains (nchains = 4) with a sampling frequency of 100. The first 25% of the samples were discarded (sump burnin = 2500) as determined by the stationarity of lnL assessed with Tracer v. 1.4 [35]; the remaining trees were used to construct the 50% majority-rule consensus tree and to estimate the nodal support as posterior probability values [36]. Genetic distances (uncorrected p-distance) were calculated with MEGA v5.

## Results

### Morphological identification of infections in natural snail populations

We found larval stages of *Echinostoma* spp. in the snail populations sampled in three of the seven reservoirs in the River Ruhr drainage in Germany and in two of the five lakes in Iceland (see Table 2 for details on hosts and localities). Three lymnaeid snail species acted as first intermediate hosts of *Echinostoma* spp. of the ‘*revolutum*’ species complex in the areas studied: *R. peregra* in the lakes in Iceland and *R. auricularia*, *R. peregra* and *S. palustris* in the reservoirs in Germany. Prevalences were usually low (typically 1-3%) but occasionally higher values were registered (Table 2).

**Table 2:** **Prevalence of *Echinostoma *spp. from natural infections in *Radix *spp. and *Stagnicola palustris *in Germany and Iceland**

**Species**	**Host**	**Locality**	**Prevalence (%)**
***Echinostoma revolutum***	*Radix peregra*	Lake Myvatn (Iceland)	2.31
	*Radix auricularia*	Hennetalsperre (Germany)	1.92 - 10.00
	*Radix peregra*	Hennetalsperre (Germany)	37.50^a^
	*Stagnicola palustris*	Hengsteysee (Germany)	0.74
***Echinostoma *sp. IG**	*Radix peregra*	Nordic House (Iceland)	0.94
	*Radix auricularia*	Baldeneysee (Germany)	1.32 (2009)^b^
	*Radix auricularia*	Hengsteysee (Germany)	2.00 - 2.90 (2009)^b^
	*Radix auricularia*	Hengsteysee (Germany)	1.56 (2011)^b^

^a^ Sample size small (n = 16); ^b ^Year indicated for different surveys of the same snail host.

Values are calculated for homogenous distinct samples only.

**Figure 1:**
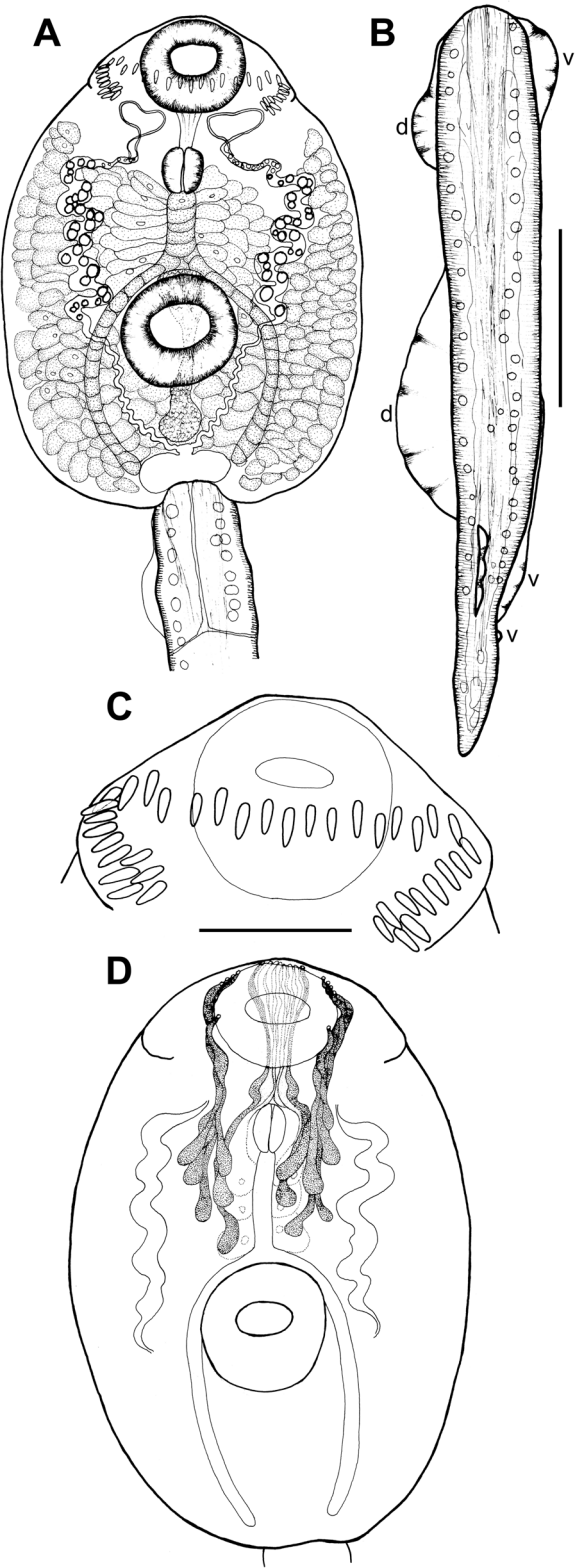
***Echinostoma *sp. IG, drawings of live cercaria. A**. Body, ventral view. **B**. Tail, lateral view (note that only one of the two ventro-lateral fin-folds is illustrated). **C**. Head collar. **D**. Schematic illustration of the para-oesophageal gland-cells. *Abbreviations*: d, dorsal fin-fold; v, ventral fin-fold. *Scale-bars*: **A**, **B**, 100 μm; **C**, 50 μm.

**Figure 2:**
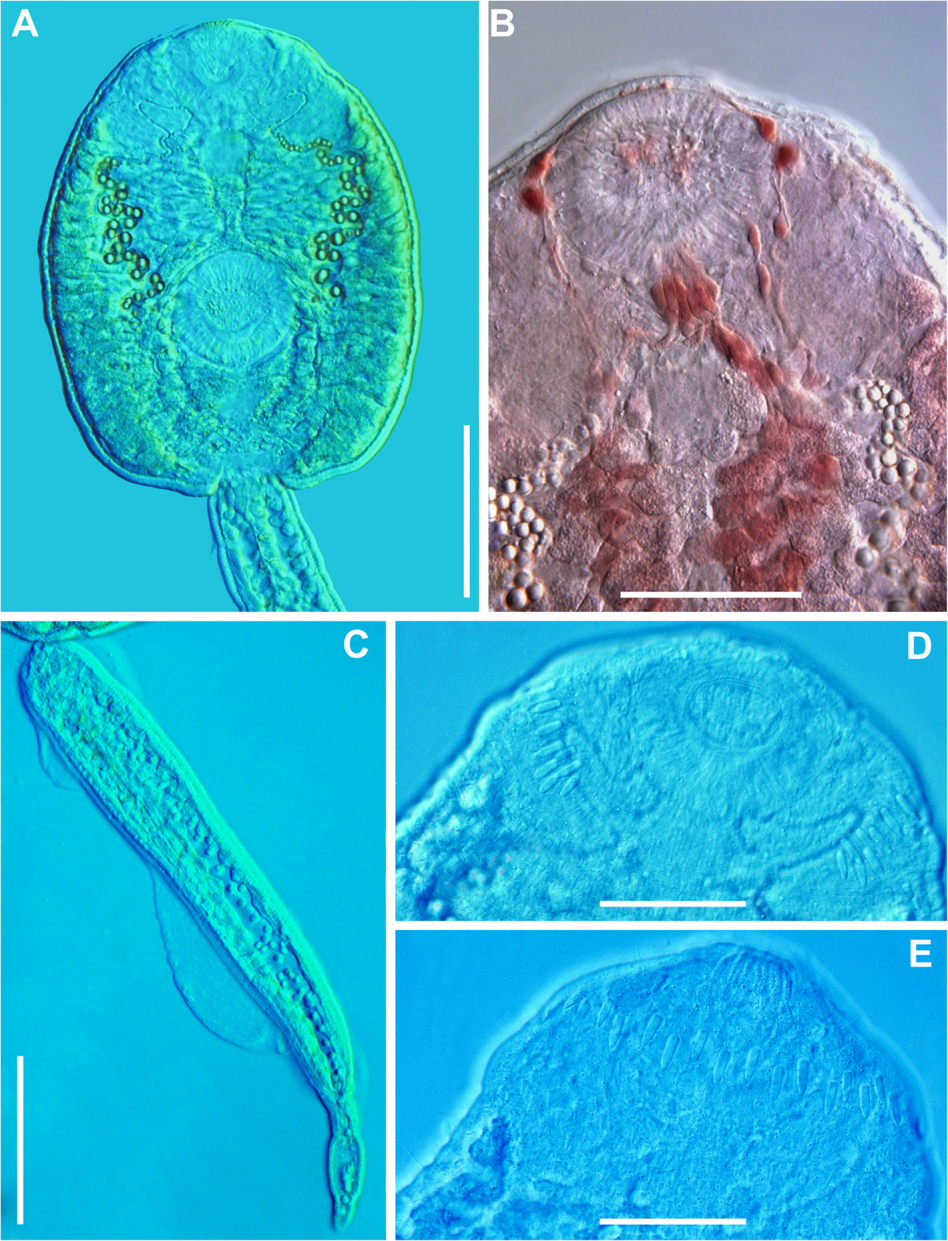
***Echinostoma *sp. IG, microphotographs of live cercaria. A**. Body, ventral view. **B**. Dorsal view showing para-oesophageal gland-cells and outlets (staining with Neutral Red) **C**. Tail, lateral view. **D**. Head collar, ventral view showing angle and lateral spines. **E**. Head collar, dorsal view, showing dorsal collar spines. *Scale-bars*: **A**, **C**, 100 μm; **B**, **D**, **E**, 50 μm.

**Figure 3:**
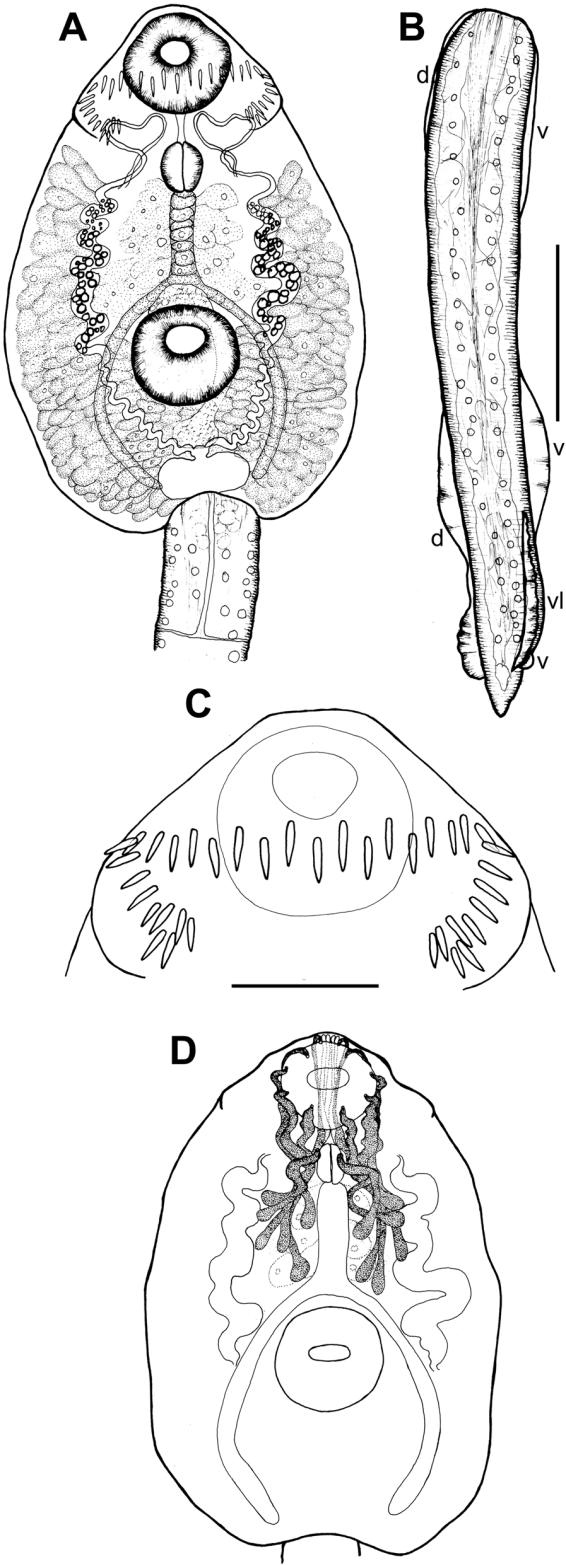
***Echinostoma revolutum*, drawings of live cercaria. A**. Body, ventral view. **B**. Tail, lateral view (note that only one of the two ventro-lateral fin-folds is illustrated). **C**. Head collar. **D**. Schematic illustration of the para-oesophageal gland-cells. *Abbreviations*: d, dorsal fin-fold; v, ventral fin-fold; vl, ventro-lateral fin-fold. *Scale-bars*: **A**, **B**, 100 μm; **C**, 50 μm.

**Figure 4:**
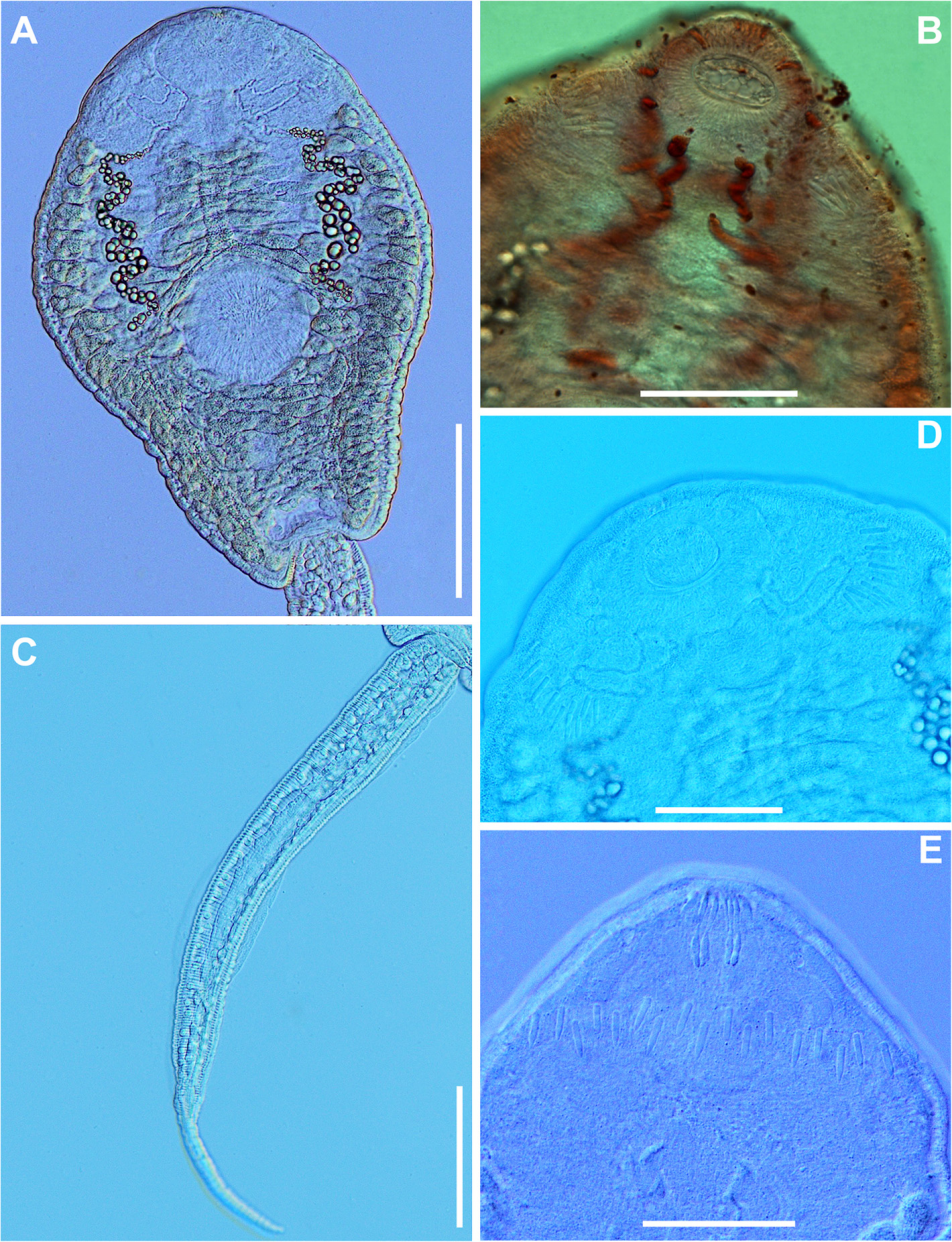
***Echinostoma revolutum*, microphotographs of live cercaria. A**. Body, ventral view. **B**. Ventral view showing outlets of para-oesophageal gland-cells (staining with Neutral Red). **C**. Tail, lateral view. **D**. Head collar, ventral view showing angle and lateral spines. **E**. Head collar, dorsal view showing dorsal collar spines. *Scale-bars*: **A**, **C**, 100 μm; **B**, **D**, **E**, 50 μm.

**Figure 5:**
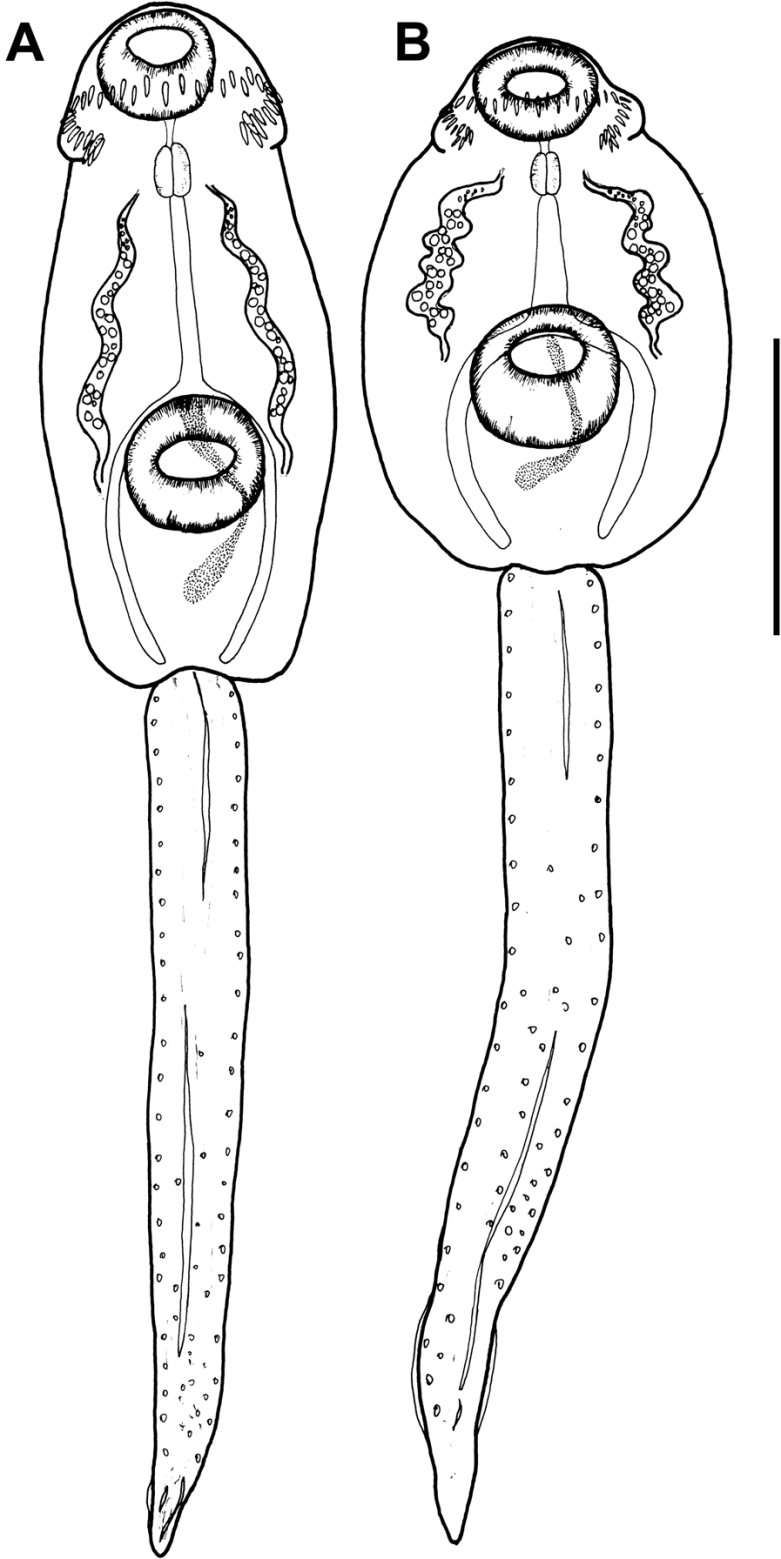
**Cercariae fixed in 4% formaldehyde solution. A**. *Echinostoma *sp. IG. **B**. *Echinostoma revolutum*. *scale-bar*: 100 μm.

Detailed examination of cercarial morphology allowed us to identify two types of echinostomatid cercariae among the isolates sampled in Iceland and Germany (Figures 1, 2, 3, 4, 5). Both types belong to the ‘*revolutum*’ species complex of *Echinostoma* which is characterised by the following features of the cercariae: (i) 37 collar spines with an arrangement 5-6-15-6-5 (5 angle and 6 lateral spines on each side and 15 dorsal spines in a double row; Figures 1C, 2D,E, 3C, 4D,E); (ii) tail with a tip forming a highly contractile attenuated process and seven prominent tegumental fin-folds (2 dorsal, 3 ventral and 2 ventro-lateral; Figures 1B, 2C, 3B, 4C); and (iii) a flame-cell formula 2[(3 + 3 + 3) + (3 + 3 + 3)] = 36.

Eleven isolates (three ex *R. peregra* from Iceland, plus two ex *R. auricularia*, five ex *R. peregra* and one ex *S. palustris* from Germany) were identified as *E. revolutum* based on cercarial morphology and especially the presence of 12 small para-oesophageal gland-cells with long ducts, located between pharynx and ventral sucker [24] (Figures 2B, 4B). However, seven isolates of cercariae, one ex *R. peregra* from Iceland and six ex *R. auricularia* from Germany, further referred to as *Echinostoma* sp. IG (indicating the origin of the isolates *i.e.* Iceland and Germany) exhibited slight differences from the isolates identified as *E. revolutum* as follows: (i) collar spines with blunt (Figures 1C, 2D,E) *vs* sharp (Figures 3C, 4D,E) tips; (ii) para-oesophageal gland-cell outlets opening at the margin of the oral sucker only (one dorsal pair, four dorsolateral pairs, and one ventro-lateral pair; see Figures 1D, 2B) *vs* openings present on the ventral surface of the body (one pair at the level of pharynx; the remaining *i.e.* one dorsal pair, one dorsolateral pair, and three ventro-lateral pairs opening at the margin of the oral sucker, see Figures 3D, 4B); and (iii) distal dorsal tail fin-fold large *vs* less prominent (length 40-60% of tail length *vs* 20-38%; width c.70% of tail width *vs* 20-30%; compare Figures 1B, 2C and 3B, 4C; Table 3). Comparison of the metrical data obtained for live cercariae revealed that *Echinostoma* sp. IG had a shorter tail, with distinctly larger distal dorsal fin-fold and shorter distal ventral fin-fold (Table 3). Furthermore, although it was difficult to observe the fin-folds in fixed material thus rendering differentiation difficult, the cercariae of *Echinostoma* sp. IG were characterised by a distinctly more elongate, narrower body and a shorter tail (Figure 5; Table 3); this represents another distinguishing feature for the two European species studied by us.

**Table 3:** **Comparative metrical data (in μm) for live and fixed cercariae of *Echinostoma *sp. IG and *E. revolutum *from natural infections in *Radix *spp. and *Stagnicola palustris *in Germany and Iceland**

**Species**	***Echinostoma *sp. IG**			***E. revolutum***		
	**Live material**	**Fixed material**		**Live material**	**Fixed material**	
	**Range**	**Range**	**Mean**	**Range**	**Range**	**Mean**
**Body length**	260 **-** 362	228 **-** 292	254	303 **-** 427	159 **-** 234	188
**Body width (max.)**	184 **-** 249	90 **-** 97	94	193 **-** 251	107 **-** 125	112
**Oral sucker length**	45 **-** 63	36 **-** 46	42	56 **-** 71	38 **-** 52	45
**Oral sucker width**	50 **-** 66	37 **-** 45	42	53 **-** 68	37 **-** 49	42
**Ventral sucker length**	54 **-** 72	43 **-** 54	48	63 **-** 83	47 **-** 66	55
**Ventral sucker width**	57 **-** 81	44 **-** 47	46	58 **-** 83	48 **-** 60	54
**Pharynx length**	25 **-** 29	16 **-** 25	20	27 **-** 36	20 **-** 24	21
**Pharynx width**	22 **-** 26	12 **-** 19	15	25 **-** 29	13 **-** 14	13
**Oesophagus length**	56 **-** 89	61 **-** 96	78	54 **-** 103	30 **-** 55	40
**Tail length**	334 **-** 353	296 **-** 378	344	364 **-** 417	316 **-** 405	367
**Tail width (at base)**	44 **-** 49	30 **-** 34	32	39 **-** 52	20 **-** 36	27
**Tail-tip length**	67 **-** 83	**-**	**-**	35 **-** 93	**-**	**-**
**Proximal dorsal fin-fold length**	49 **-** 63	50	**-**	41 **-** 153	**-**	**-**
**Proximal dorsal fin-fold width**	14 **-** 15	**-**	**-**	5 **-** 13	8 **-** 11	9
**Distal dorsal fin-fold length**	147 **-** 212	106 **-** 154	120	72 **-** 159	**-**	**-**
**Distal dorsal fin-fold width**	30 **-** 35	14 **-** 21	16	7 **-** 16	**-**	**-**
**Proximal ventral fin-fold length**	47 **-** 90	73	**-**	51 **-** 116	85	**-**
**Proximal ventral fin-fold width**	12 **-** 15	**-**	**-**	4 **-** 6	5	**-**
**Distal ventral fin-fold length**	44 **-** 64	41	**-**	74 **-** 202	99 **-** 157	125
**Distal ventral fin-fold length**	6 **-** 18	8	**-**	7 **-** 14	**-**	**-**

### Molecular analysis

A total of 14 partial *nad*1 sequences was generated (11 for *E. revolutum* and 3 for *Echinostoma* sp. IG; Table 1). These sequences were aligned with selected published sequences representing the data available for eight species of the ‘*revolutum*’ species complex of *Echinostoma* generated from both laboratory strains [13] and natural isolates [9,11,14]; two otherwise unpublished sequences were also retrieved from GenBank (see Table 1 for details). The aligned dataset included 39 sequences and was comprised of 472 nt positions after trimming the ends to match the shortest aligned sequences. Sequences for *Echinoparyphium* spp. of Kostadinova *et al.*[14] were used as outgroups (Table 1).

**Figure 6:**
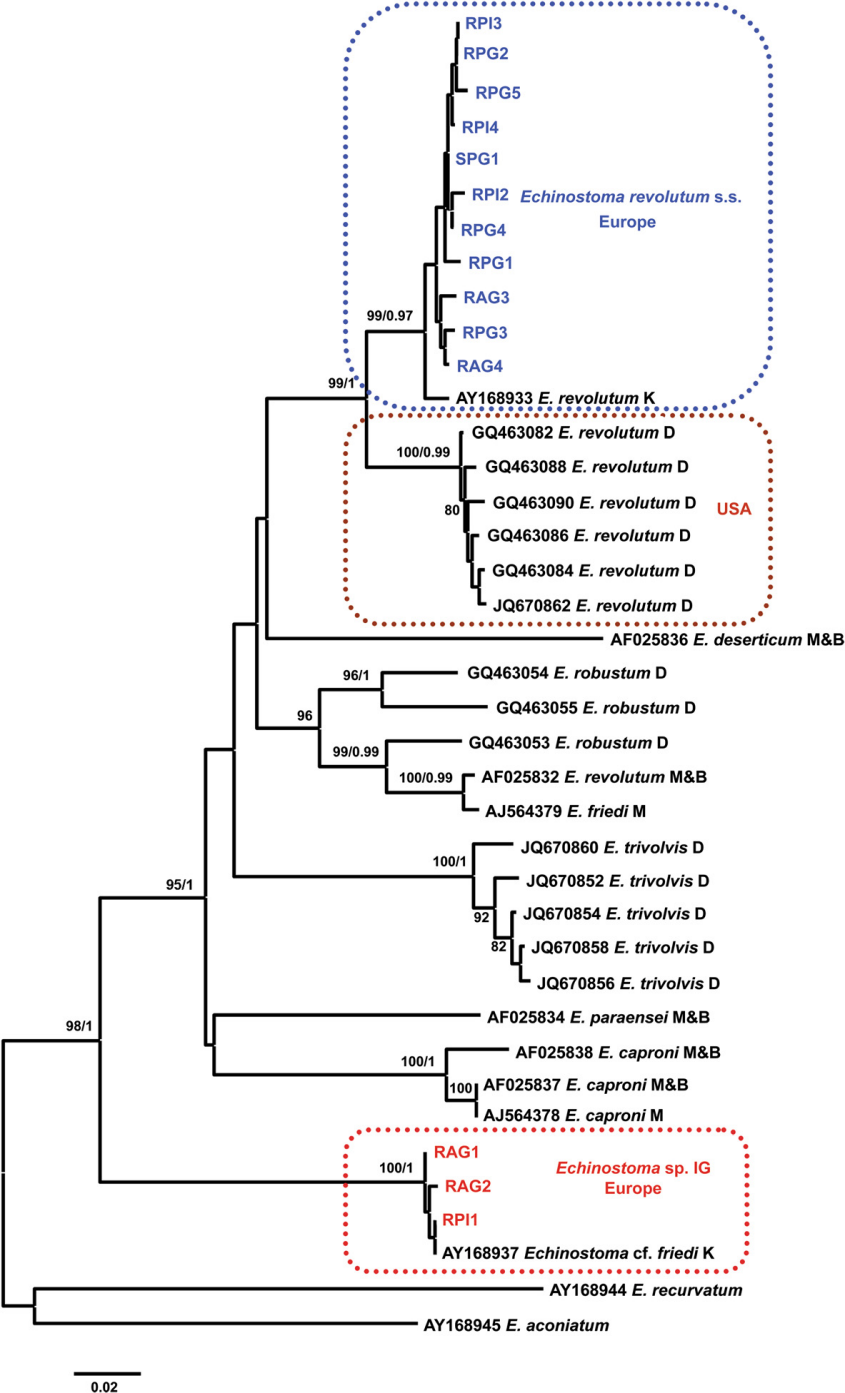
**Neighbour-joining (NJ) phylogram reconstructed using the newly-generated and retrieved from GenBank *nad1 *sequences (472 nt positions) for *Echinostoma *spp. of the ‘*revolutum*’ species complex. **Outgroup: *Echinoparyphium *spp*.* Nodal support (bootstrap values > 70% shown only) inferred from 1,000 replicates; these are followed by posterior probabilities from BI analysis. The scale-bar indicates expected number of substitutions per site. Sequence identification as in GenBank followed by a letter: D, Detwiler et al. [9,11]; K, Kostadinova *et al.*[14]; M, Marcilla *et al.* (GenBank); M & B, Morgan & Blair [7,13].

Both NJ and BI analyses resulted in consensus trees with similar topologies (see Figure 6 for a phylogeny inferred from genetic distances and BI). The newly-generated sequences for *E*. *revolutum* formed a strongly supported clade which included a sequence for *E. revolutum* (s.s.) of Kostadinova *et al.*[14] (see also [6]). On the other hand, the sequences for the isolates identified as *Echinostoma* sp. IG formed a strongly supported reciprocally monophyletic lineage, basal to *Echinostoma* spp., which also incorporated the sequence for an isolate from Wales (UK) provisionally identified as *Echinostoma* cf. *friedi* by Kostadinova *et al.*[14]. The isolates comprising this lineage also exhibited the highest levels of divergence from the isolates of *Echinostoma* spp. analysed (p-distance range 17.2-21.6%; divergence from *E. friedi* (AJ564379) (p-distance range 18.9-19.1%).

Unexpectedly, the European isolates of *E. revolutum* and those obtained from natural infections in *Lymnaea elodes* and *Ondatra zibethicus* (L.) in North America by Detwiler *et al.*[11] formed two strongly supported sister lineages. This solution (both NJ and BI analyses) was consistent with the distinctly higher inter-lineage divergence (p-distance; 4.9–6.8%) compared with intra-lineage divergence (p-distance range, European isolates: 0–2.1%, North American isolates: 0.4–1.1%). These data indicate that the North American isolates represent another cryptic species of the ‘*revolutum*’ species complex.

Another unexpected result was that the sequence for *Echinostoma revolutum* of Morgan and Blair [7,13] (AF025832; isolate from Europe) exhibited a strong association with the sequence for *Echinostoma friedi* of Marcilla *et al*. (unpublished, GenBank AJ564379) based on an isolate of this species recently described by these authors [22] from Spain (p-distance 0.8%; divergence from nearest neighbours, *i.e. Echinostoma robustum* sensu Detwiler *et al.*[11], of 4.9-9.1%. The clade comprising the former two European isolates and those of *E. robustum* from North America exhibited a complex structure suggesting the existence of at least three species (subclade support indicated in Figure 6).

## Discussion

The combined morphological and DNA-based approaches in this first intensive screening of *Radix* spp. for infections with *Echinostoma* spp. allowed us to delineate two cryptic species of the ‘*revolutum*’ complex in central and northern Europe. Furthermore, comparative sequence analyses depicted three additional cryptic lineages in North America.

Both distance- and model-based phylogenies provided high support for reciprocal monophyly of *Echinostoma* sp. IG. The isolates of this lineage, that evidently represents a new species, awaiting further formal description after a discovery of the adult parasite stage, were found to be clearly distinguishable among the European isolates by using both morphological and molecular evidence. Although the identification of the European isolates of *Echinostoma* spp. followed the standard taxonomic practice, the detection of the new cryptic species required substantial taxonomic expertise. This involved detailed knowledge on the variation of the features used for species delimitation based on thorough morphological examination of a large number of cercariae from each isolate. The corroboration of our hypothesis for the distinct species status of the two species of *Echinostoma* parasitising snail populations in Germany and Iceland on the basis of molecular data thus may appear secondary.

However, the distinguishing features are difficult to detect and/or subject to variation (reviewed in Kostadinova and Gibson [6]). For example, Kanev [3] described 16 ducts and pores of para-oesophageal gland-cells in the cercariae of *E. revolutum* ex *Lymnaea stagnalis*; of these, 12 were located on the oral sucker and four on the ventral surface. On the other hand, we detected only 12 small para-oesophageal gland-cells in the cercariae of *E. revolutum* ex *Radix* spp.; Faltýnková *et al.*[24] also provided this number for *E. revolutum* ex *L. stagnalis*. It is worth noting that recent field studies indicate that *E. revolutum* most commonly occurs in *L. stagnalis* in Europe [23,24], infections with this species have occasionally been reported in the past from *R. auricularia*, *R. peregra* and *R. ovata* (Draparnaud, 1805) [22,37-45]. Further molecular study would reveal whether *Echinostoma* spp. of the ‘*revolutum*’ species complex parasitising *L. stagnalis* and *Radix* spp. are conspecific or represent as yet undiscovered cryptic species. We believe that ‘reciprocal illumination’ *sensu* Hennig [46] of morphological characters upon a molecular-based species delimitation has a strong potential for delineating species boundaries within the ‘*revolutum*’ complex of cryptic species.

*Echinostoma* sp. IG was found to be conspecific with an isolate from Wales (UK) provisionally identified as *Echinostoma* cf. *friedi* by Kostadinova *et al.*[14]. The lineage comprising this and the newly-sequenced isolates occupied a basal position (as in Kostadinova *et al.*[14]) and this is in sharp contrast with the phylogenetic solution based on *nad*1 gene of Detwiler *et al.*[11]. These authors wrote that “A comparison of samples identified as *E. robustum* (U58102) and *E. friedi* (AY168937) reveals that they are found within the same monophyletic clade and thus do not qualify as distinct species according to a phylogenetic definition. Additionally, they are genetically similar (0.009 genetic divergence, ND1 …” and concluded that “the sample tentatively identified as *E. friedi* in Kostadinova *et al.* (2003) is genetically very similar to *E. robustum*”. Our results clearly indicate that the sequence for *E. friedi* from its type-locality in Spain (AJ564379; Marcilla *et al.* unpublished sequence in GenBank) and for the European isolate labelled as *E. revolutum* (AF025832) of Morgan and Blair [7,12,13] represent conspecific isolates; the genetic divergence between these two isolates was 0.8%, *i.e.* substantially lower than that (*i.e.* 18.9-19.1%) between the lineage containing *E*. cf. *friedi* (AY168937) of Kostadinova *et al.*[14] and the European isolate labelled as *E. revolutum* (*nad*1 sequence AF025832; ITS sequence U58102) by Morgan and Blair [7,12,13]. We believe, therefore, that Detwiler *et al.*[11] have in fact used the otherwise unpublished sequence for *E. friedi* of Marcilla *et al.* (AJ564379) but have mislabelled it (as AY168937).

Kostadinova *et al.*[14] indicated a tentative affiliation to *E. robustum* of the isolates of the ‘Australian-German’ clade of *Echinostoma* spp. of Morgan and Blair [7], but suggested that this specific identification is pending a redescription of both larval and adult stages. The present results indicate that suggesting synonymy for the European isolate studied by Morgan and Blair [7,12,13] and *E. friedi* should await examination of a larger number of molecularly characterised natural isolates of the European species of the ‘*revolutum*’ complex since our knowledge on cryptic diversity in this group is still limited. This suggestion is supported by the discovery of two genetically distinct, geographically separated lineages of *E. revolutum*: *E. revolutum* s.s. from Europe and *E. revolutum* of Detwiter *et al.*[11] from North America, thus demonstrating that the suggestion for the cosmopolitan distribution of this species [11] appears to be a result of cryptic variation. Indeed, these authors noted that their results of network analyses indicate gene flow and population expansion within North America but not on a global scale. The taxonomy of the North American species can be further scrutinised using the morphological data available for cercariae and/or experimentally developed adults [11,47].

## Conclusion

The results of our study suggest that further analyses of patterns of interspecific variation based on a combination of molecular and well-documented morphological data would enhance the re-evaluation of the species and advance our understanding of the relationships within the ‘*revolutum*’ group of *Echinostoma*.

## Competing interests

The authors declare that they have no competing interests.

## Authors’ contributions

CS, MS and KS obtained the samples. CS, AF, MS and SG undertook the morphological study. SG carried out the sequencing and phylogenetic analysis. CS, SG, AF and MS prepared the first draft of the MS. KS, BS and AK conceived and coordinated the study and helped to draft the MS. All authors read and approved the final manuscript.
